# Estimation of the Inhaled Dose of Airborne Pollutants during Commuting: Case Study and Application for the General Population

**DOI:** 10.3390/ijerph17176066

**Published:** 2020-08-20

**Authors:** Francesca Borghi, Giacomo Fanti, Andrea Cattaneo, Davide Campagnolo, Sabrina Rovelli, Marta Keller, Andrea Spinazzè, Domenico Maria Cavallo

**Affiliations:** Department of Science and High Technology, University of Insubria, 22100 Como, Italy; andrea.cattaneo@uninsubria.it (A.C.); davide.campagnolo@uninsubria.it (D.C.); sabrina.rovelli@uninsubria.it (S.R.); mkeller@studenti.uninsubria.it (M.K.); andrea.spinazze@uninsubria.it (A.S.); domenico.cavallo@uninsubria.it (D.M.C.)

**Keywords:** pollution, PM, commuting, travel mode, active transportation, micro-environment, risk assessment, pulmonary ventilation rate

## Abstract

During rush hours, commuters are exposed to high concentrations and peaks of traffic-related air pollutants. The aims of this study were therefore to extend the inhaled dose estimation outcomes from a previous work investigating the inhaled dose of a typical commuter in the city of Milan, Italy, and to extend these results to a wider population. The estimation of the dose of pollutants inhaled by commuters and deposited within the respiratory tract could be useful to help commuters in choosing the modes of transport with the lowest exposure and to increase their awareness regarding this topic. In addition, these results could provide useful information to policy makers, for the creation/improvement of a mobility that takes these results into account. The principal result outcomes from the first part of the project (case study on a typical commuter in the city of Milan) show that during the winter period, the maximum deposited mass values were estimated in the “Other” environments and in “Underground”. During the summer period, the maximum values were estimated in the “Other” and “Walking (high-traffic conditions)” environments. For both summer and winter, the lowest values were estimated in the “Car” and “Walking (low-traffic conditions)” environments. Regarding the second part of the study (the extension of the results to the general population of commuters in the city of Milan), the main results show that the period of permanence in a given micro-environment (ME) has an important influence on the inhaled dose, as well as the pulmonary ventilation rate. In addition to these results, it is of primary importance to report how the inhaled dose of pollutants can be strongly influenced by the time spent in a particular environment, as well as the subject’s pulmonary ventilation rate and pollutant exposure levels. For these reasons, the evaluation of these parameters (pulmonary ventilation rate and permanence time, in addition to the exposure concentration levels) for estimating the inhaled dose is of particular relevance.

## 1. Introduction

The association between traffic-related air pollution and health is well recognized and reported in the literature, from both epidemiological and toxicological studies [1]: these chemical factors may affect human health, especially in urban areas, representing hotspots of traffic emissions. In particular, exposure to air pollutants in traffic environments has been related to long- and short-term cardiovascular and respiratory effects [2]. During rush hours, commuters are exposed to high concentrations of traffic-related air pollutants [3], usually exceeding air quality standards [4]. Moreover, commuting in rush hours may have the potential to disproportionately contribute to daily exposures, despite the time spent in them being reduced on average to 1.5–2 h per day [4,5,6]. For these reasons, many studies have been conducted in several cities: the results generally show that motorists and public transport commuters are exposed to higher pollutant levels than cyclists and pedestrians [7]. Contrariwise, due to the high pulmonary ventilation rate measured in active commuting, cyclists and pedestrians may inhale a higher dose of pollutants, despite lower exposure [8]. In recent years, it has been suggested that assessing the health impact in transport micro-environments (MEs) by only considering the exposure to environmental pollutant concentrations is not entirely representative of personal exposure: the use of the inhaled pollutant dose may be one of the most interesting parameters to explore to complete the fundamental information brought by exposure assessment.

The aims of this study were therefore to further elaborate, using the multiple-path particle dosimetry model for the estimation of the deposited particulate matter (PM) mass in the different regions of the respiratory tract (i.e., head, tracheobronchial and pulmonary [9]) and extending the results to the general commuter population of Milan, the results obtained in a previous study [10] investigating the exposure to airborne pollutants and the inhaled dose of a typical commuter in the city of Milan, Italy. The objective was to extend the results to a wider population (commuters within the Milan metropolitan area, one of the most polluted across Europe); for this purpose, the exposure levels measured in the breathing zone of a typical commuter were associated with the average residence times spent within the various transit MEs by the evaluated population.

Briefly, the previous study [10], on which this work is based, aimed to evaluate the exposure of commuters to different pollutants (nitrogen dioxide (NO_2_) and fractionated particulate matter (PM), including ultrafine particles (UFPs)) using miniaturized and portable real-time monitoring instruments in selected MEs. In particular, measurements were performed along a typical commuter route, considering different traffic and non-traffic MEs. Principal results show that higher exposure levels were measured in Underground (for all PM fractions and NO_2_) and in the Car (UFP), while lower exposure levels were measured in Car (PM and NO_2_) and in Train (UFP).

The present study was therefore performed to evaluate in greater depth the issue of the pollutants inhaled dose in different MEs, first investigating the deposition of different fractions of PM in the respiratory tract, and then extending the results to the general population of Milan.

## 2. Materials and Methods

### 2.1. Study Design and Instrumentation

**Figure 1:**
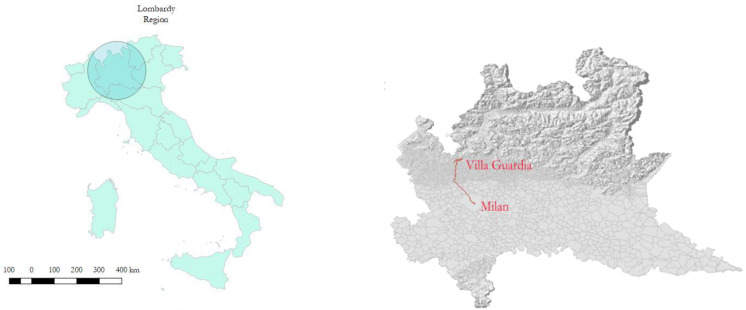
Lombardy region (**left**) and the complete route traveled by the subject from Villa Guardia (45°47′ N 9°01′ E) to the city center of Milan (45°27′ N 9°11′ E) (**right**).

This study was based on data collected during a monitoring campaign conducted in winter and summer 2019, the methods of which are presented elsewhere [10]. Briefly, to simulate a typical home-to-work (and return) commuter route, a fixed route was defined a priori from a Lombardy provincial city to the Milan city center, the largest city in the region and one of the most populous metropolitan cities in Europe (Figure 1).

With the intent to analyze (i) the exposure concentration and the (ii) dose of selected pollutants inhaled by the subjects (and to estimate the dose inhaled by the general population) in different transit MEs typically frequented by commuters, the environments were divided as follows: Walking (in low-traffic (LT) and high-traffic (HT) conditions), Bike, Car, Underground, Train, Indoor (office), and Other MEs (defined as the transition period (2 min) while moving from one environment to another). Car ventilation (e.g., ventilation intensity, windows closed) was maintained in constant conditions during all journeys [11]. The residence times (min) and the route length (km) of the different MEs are reported in Table 1.

The continuous determination of size-fractionated PM (PM_1_, PM_2.5_, PM_4_, and PM_10_) concentrations was performed using a portable direct-reading monitor (Aerocet 831-Met One Instrument Inc., Grant Pass, Oregon, USA), worn by one of the authors (G.F.) using a backpack. PM_2.5_ samples were also collected using a GK2.05 sampler (BGI Inc., Waltham, MA, USA), operated with a sampling pump with a flow rate equal to 4 L/min; particles were collected using polytetrafluoroethylene filters. The mass concentration was determined by gravimetric analysis following a standard reference method [12,13] and previous studies [14,15,16]. Gravimetric data were used to correct the PM data acquired via the direct-reading instrument by calculating a daily correction factor applied a posteriori to the whole PM dataset [17].

### 2.2. Estimation of the Inhaled Dose

In this study, the estimation of the inhaled doses of different PM fractions for (i) a selected subject and for (ii) the general commuter population in the city of Milan was performed. The dose estimation for the selected subject (in good physical condition and aged 30 years) study was carried out using the MPPD V.3.04 (multiple-path particle dosimetry) [18] model, using the Yeh–Shum symmetric model for humans. The default physiological parameters (breathing frequency: 12 breaths/min; tidal volume: 625 mL; inspiration fraction: 0.5; pause fraction: 0) were entered for the model computation. The deposition fraction in the respiratory tract (reported for the pulmonary, tracheobronchial, and upper airways, as well as the total) was used to estimate the PM mass (µg) inhaled by the subject, following Equation (1):

Deposited mass: DF × C × t × V(1)

Equation (1). Estimation of the inhaled dose (µg). DF: deposition fraction (estimated via MPPD V.3.04 model); C: exposure concentration (µg/m^3^) (measured during the monitoring campaign); t: time spent in a particular ME (h) (registered using a time activity diary); V: subject minute ventilation (m^3^/h) (measured during the monitoring campaign).

Equation (2) was used to estimate the dose inhaled by the general commuter population [19]:

Inhaled Dose: C × t × VE(2)

Equation (2). Inhaled dose estimation (µg). C: exposure concentration (µg/m^3^); t: time spent in a particular ME (min); VE: pulmonary ventilation rate (m^3^/min).

In this study, Equation (2) was used to estimate the dose inhaled by the general population (according to gender, time spent in a particular ME, ME, moment of the day and season) while commuting in different transit MEs. In particular, the exposure concentration data refer to those acquired in the case study [10], the values of residence times (15, 30, 30 and 90 min), as well as the MEs visited by the subject and the gender, were acquired from the most recent Italian census (ISTAT—Istituto Nazionale di Statistica (2011), available at [20]), while the pulmonary ventilation rates, selected for women and men, refer to values reported in the literature [21]. In particular, “light activity levels” were selected for passive commuting (38.2 ± 2.4 L/min and 31.0 ± 4.1 L/min for men and women, respectively) and “moderate activity levels” for active commuting, such as cycling and walking (73.5 ± 4.8 L/min and 63.7 ± 7.7 L/min for men and women, respectively). The inhalation dose data were also processed according to the period of the day (morning: to work/evening: homeward) and to the season (summer/winter), starting from the exposure data obtained from the monitoring campaign.

For the calculations, the commuting period results from the ISTAT database were selected by considering the most similar commuting period (8:15–9:15 a.m.) to the study design, applied also for the evening return to home and for both the summer and winter periods (even if the commuting patterns could change over seasons).

Data were analyzed using the Statistical Package for the Social Sciences Statistic version 20.0 (IBM, Armonk, NY, USA), and a significance level of 0.05 was used in all statistical tests.

## 3. Results and Discussions

### 3.1. Case Study

Table 2 reports the mass (µg) of size-fractionated PM deposited in different sections of the respiratory tract, as a function of the season.

**Figure 2:**
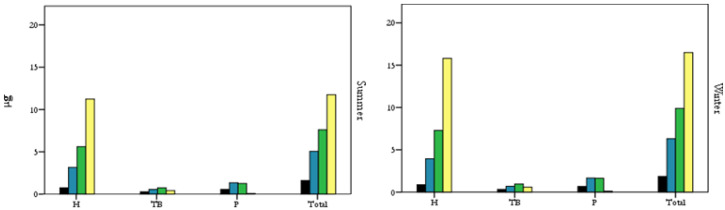
PM deposited mass (µg) in the respiratory tract (H: head; TB: tracheobronchial; P: pulmonary; total: H + TB + P). Black: PM_1_; Blue: PM_2.5_; Green: PM_4_; Yellow: PM_10_.

Figure 2 shows the PM mass (µg) deposited in the respiratory tract, estimated for the summer and winter periods. As reported in the figure, the PM deposited mass was higher during the winter period for all PM fractions, even if the differences between the estimates for summer and winter were minimal (<1 µg for PM_1_, PM_2.5_, and PM_4_; >2 µg for PM_10_). Moreover, the mass deposited in the upper airways (H) contributed significantly to the mass deposited in the whole airways (total) for both summer and winter (47% for PM_1_, 62% for PM_2.5_, 74% for PM_4_, and 96% for PM_10_, on average).

Regarding the estimation of the PM deposited mass as a function of the ME visited by the commuter, as reported in Table 3, and considering the total mass deposited in the entire respiratory tract, for the winter period the maximum values were estimated in the “Other” environments and in “Underground”, for all the PM fractions considered, followed by the “Indoor” and the “Walking (LT)” environments. The lowest values were estimated in the “Car” and “Walking (LT)” environments. For the summer period, the maximum values were estimated in the “Other” and “Walking (HT)” environments. As during the winter, the lowest values were found in the “Car” and “Walking (LT) environments.

A problem stated by the scientific literature regards the lack of data to provide a systematic basis for comparing the exposure concentrations in different transportation modes, due to different sources of variability (i.e., period of the day, season, and location) [22]. As stated by the authors, indeed, transportation mode exposure concentrations can vary in accordance with these environmental factors (i.e., season and time of day), which are related to atmospheric stability and pollutant dispersion. Moreover, exposure concentration levels in different transportation modes may be affected by the traffic flow, by proximity to emissions hotspots, and by emissions from other vehicles [11,23]. For example, Frey and collaborators, in their recent paper, reported how PM_2.5_ exposure concentration levels are sensitive firstly to the mode of transport, followed by the time of the day and by the monitoring season [22].

Not considering the “Other” environment (as it is difficult to characterize, since it includes all the periods of transition while moving from one ME to another), for the winter period the highest values of PM deposited mass were estimated in the “Underground”, “Indoor”, and “Walking (HT)” environments. Although the time spent in the “Underground” environment was small (0.4 h) and the estimated subject ventilation rate was moderate (0.66 m^3^/h), this environment was characterized by the highest PM exposure concentrations [10], probably due to the presence of indoor PM sources (e.g., abrasion of rails, wheels, and brakes and resuspension of particles) [4]. Conversely, the time spent in the “Indoor” environment, due to the study design, was the highest among the investigated environments (>1.5 h). Finally, in the “Walking (HT)” environment, we measured the highest pulmonary ventilation rate values (1.30 m^3^/h); this could justify the high inhaled dose of pollutants in this environment. During the summer, the “Walking (HT)” environment was the environment characterized by the highest PM deposited mass values, due to the combination of a high subject pulmonary ventilation rate (1.30 m^3^/h) and high exposure concentration levels. During both winter and summer, the mass deposited values were lower in the “Car” and “Walking (LT)” environments; this can be justified by the reduced permanence time in these environments (<20 min for the “Walking (LT)” environment and <1 h for the “Car” environment).

These results show how the different factors taken into account for the calculation of the inhaled dose (i.e., exposure concentration, time spent in a particular environment, and lung ventilation rate) can contribute significantly to the PM deposited mass. Even if not specifically performed in this study, a sensitivity analysis was carried out by the authors in a similar study conducted in the city of Milan; the principal results show how the parameters having a major impact on the inhaled dose are the time spent in a ME and personal exposure levels. In this case, VE seems to have a low impact on the inhaled dose, both for MEs and kinds of pollutants [24].

In general, a previous study [7] suggests how the inhaled dose of pollutants is higher during active commuting compared to motorized trips: this can be explained by the subjects’ increased minute ventilation. Another study [25] indicates that, although exposure levels are low during walking trips, pulmonary ventilation rates are generally higher if compared to other MEs; for this reason, it is particularly important to consider both variables for the estimation of the inhaled dose (e.g., exposure concentrations and ventilation rate). It should be noted that the scientific literature also reports that the residence time is an important factor to consider in the inhaled dose estimation, as well as the pulmonary ventilation rate. In fact, active transport (walking and cycling) is characterized by higher exposure levels and inhaled doses of PM_2.5_ than other transport modes on a comparable trip [3,19].

### 3.2. General Population

Estimation of the pollutant inhaled dose was carried out on a commuter population that usually travels in the city of Milan using the methodology described in paragraph 2.2. The estimated values of the inhaled dose of size-fractionated PM segregated by ME, time spent commuting, and gender are reported in Table 4. These data were further subdivided according to the season (summer/winter) and the commuting period of the day (morning/afternoon). As expected, Table 4 shows how the period of permanence in each ME impacts on the inhaled dose. Furthermore, as previously discussed, higher values of inhaled doses of PM were estimated during active commuting (“Cycling” and “Walking”), due to the increased pulmonary ventilation rate. In addition, due to the lower pulmonary ventilation rate in women, it seems that women inhale a lower dose of pollutants, although there is no statistically significant difference between the inhaled doses of pollutants between women and men (*p* > 0.05 for all PM fractions; Mann–Whitney *U* test, performed after checking the normality—resulting neither normally not log-normally distributed—of the data distribution via Kolmogorov–Smirnov test).

Statistically significant differences (*p* < 0.05) were not found by comparing the two monitoring periods (morning/afternoon) but as expected, occurred as a function of the considered ME.

Following the literature [11], the non-parametric Kruskal–Wallis test was used to assess the differences (in terms of inhaled dose) among the MEs groups. Furthermore, pairwise post hoc Mann–Whitney tests were used to further investigate the data when the Kruskal–Wallis test results were found to be significant [26]. This test allowed the statistically significant differences to be identified within the data. However, in order to limit the Type I error rate, a Bonferroni correction was applied for each post hoc Mann–Whitney test. As such, the statistically significant value of 0.05 was divided by the number of the possible comparisons among the groups (*N* = 10). The resulting value was the critical value (p) considered in the post hoc Mann–Whitney test [26].

In detail, as reported in Table 5, statistically significant differences were found between the “Walking” environment and the other MEs. Moreover, there were no statistically significant differences between the two active transport methods (“Cycling” and “Walking”).

Further differences in the inhaled doses estimated across different MEs can also occur according to the season. In fact, during winter the differences between MEs corresponded with those of the entire study period (i.e., statistically significant differences were found between active and passive commuting); in summer, however, the only statistically significant differences were found for the ME “Walking” versus the MEs “Train” and “Car” (Table S1).

**Figure 3:**
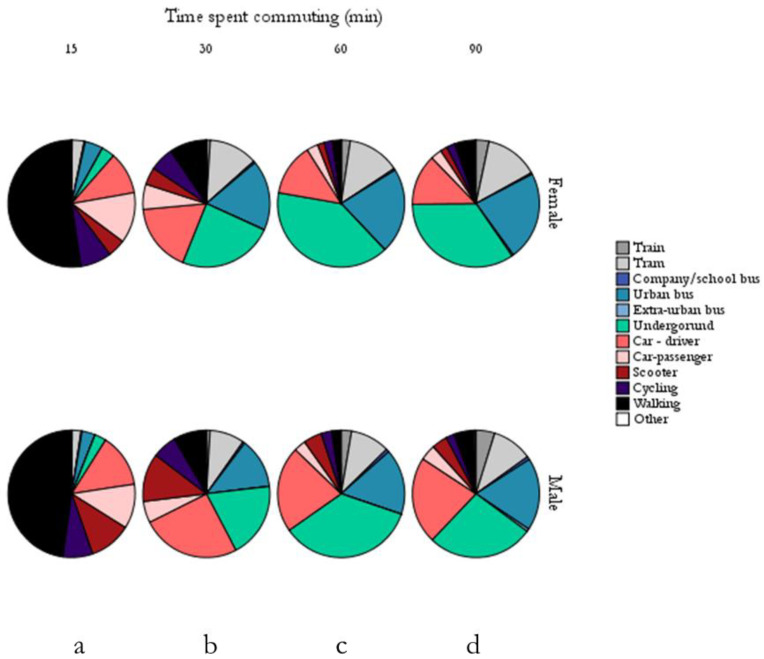
Proportions of subjects who move through different transport MEs within the city of Milan. In the figure, the data are divided by gender (female or male) and by permanence periods ((**a**): 15 min, (**b**): 30 min, (**c**): 60 min, (**d**): 90 min).

To provide a broader perspective to the study, the information obtained from the case study and from the general population analysis was associated with the average commuting periods of the general population commuting in the city of Milan. A summary of these data (ISTAT 2011) was shown in Figure 3. Although the permanence time (reported by the Italian census (ISTAT 2011 and used in this part of the study)) in a particular ME (15, 30, 60, 90 min) is different according to the gender, it is possible to notice how the preferred type of commuting is walking (52% and 48%, respectively, for women and men) for short trips (15 min—Figure 3a), followed by commuting by car (24% and 25%, respectively, for women and men) and cycling (8% for both genders). Public transport is not generally chosen for short trips (<15 min). Compared to the 15 min periods, the number of subjects who choose to travel by bike for 30 min (Figure 3b) is reduced to 6% for both women and men. On the contrary, the number of commuters walking for a period of >15 min decreases (9% and 8% for periods of 30 min (Figure 3b) for women and men, respectively, 2% for periods of 60 min (Figure 3c), and 5% for periods of 90 min (Figure 3d), for both genders) while, as expected, the use of public transport (metro and buses) increases with increasing commuting times (Figure 3c,d).

The analysis of this kind of information is important to consider, especially regarding the estimation of the inhaled dose in active commuting patterns (walking and cycling), as these are preferred to passive commuting for short trips. As reported before, the inhaled dose can be strongly influenced by the time spent in a particular ME and by the subject’s pulmonary ventilation rate. In fact, active transport is thus characterized by a higher inhaled dose of pollutant, if compared with the typical passive means of transport, due to (i) the higher pulmonary ventilation rate of the subjects and to (ii) the longer period of time spent in these kinds of environments. As said, although these aspects have now been consolidated, it is still difficult to define a trend in the study of the commuters’ inhaled dose of pollutants applicable to different urban contexts, since, in addition to environmental (i.e., concentrations of pollutants), micro-environmental, and personal (i.e., physiological parameters) variability, it is necessary to consider population mobility patterns (in turn influenced by different aspects, such as the urban layout). All these aspects can therefore contribute in defining the inhaled dose of airborne pollutants and should be considered for the personal and community choice of the best solution for urban commuting, in terms of the potential impact on health. For example, in the specific case of the city of Milan (information about mobility in the city of Milan is available in a recent study [27]), it is possible to note that active commuting is typically chosen for the quickest routes (15 min of travel). Therefore, direct comparisons with other studies are not possible; furthermore, this suggests that each specific case should be assessed.

### 3.3. Limits of the Study and Future Developments

This study has several limitations: (i) the inhaled doses of pollutants were estimated along a route established a priori, which although was intended to best simulate the path of an average commuter, might not be fully representative of the entire population. Moreover, these results cannot be extended to other urban areas: in fact, the concentrations of pollutants measured in different MEs and the estimation of the inhaled dose are intrinsically characterized by a high variability, especially in urban areas. Geostatistical analyses for the description of the selected route (i.e., the analysis of the population density, land use, etc.) were not conducted. In addition, (ii) the study was carried out considering a single subject, estimating the personal pulmonary ventilation rate, certainly not representative of the entire population. Moreover, (iii) due to the study design, the evening trip (return to home) did not coincide with the evening rush times, as was done for morning commuting. Finally, it is necessary to recognize that different assumptions were used to obtain data regarding the ventilation rate and the estimated inhaled dose via the MPPD model: in this way, considering the use of different levels of approximation, it is necessary to consider the presence of an intrinsic error associated with these estimates. Moreover, the worst case (in terms of deposited mass) was considered in this study, as the clearance was not evaluated or taken into account.

For these reasons, future developments could include measures also during the evening rush hours and conducted along other routes, with the aim of improving the representativeness of this study. In addition, it would be useful to evaluate the influence of micro-environmental conditions (e.g., congested conditions) on the measurement of pollutant exposure concentrations at first and, therefore, on the estimate of the pollutant inhaled dose. Finally, the commuters’ daily exposure assessments and the contextual use of biological measurements should be considered in future studies.

## 4. Conclusions

This study was divided into two sections: (i) a case study conducted on a commuter who spends different periods of time on different means of transport and (ii) an extension of the results derived from the case study to a larger population (commuters who move within the city of Milan). The principal result outcomes from the case study show that the PM deposited mass was higher during the winter period, for all PM fractions, even if the differences between the estimates for summer and winter were minimal, and that the mass deposited in the upper airways (H) contributed significantly to the mass deposited in the whole airways (total) for both summer and winter and for all PM fractions (Figure 2). Moreover, the principal results show that during the winter period, the maximum deposited mass values were estimated in the “Other” environments and in “Underground”, for all the PM fractions considered, followed by the “Indoor” and “Walking (LT)” environments. During the summer period, the maximum values were estimated in the “Other” and “Walking (HT)” environments. For both summer and winter, the lowest values were estimated in the “Car” and “Walking (LT)” environments. Generally, the high deposited mass values during active commuting were justified by the literature since in these environments (for example, “Walking” and “Cycling”), the pulmonary ventilation rates were high if compared to those measured during passive commuting, as is the time spent in MEs. For these reasons, the evaluation of these parameters (pulmonary ventilation rate and permanence time, in addition to the exposure concentration levels) for estimating the inhaled dose is of particular relevance. Regarding the second part of the study, or, rather, the extension of the results to the general population of commuters in the city of Milan, the main results show that the period of permanence in a given ME has an important influence on the inhaled dose, as well as the pulmonary ventilation rate (Table 4). Moreover, during the winter period, statistically significant differences (*p* < 0.005) occur between the “Walking” ME and passive means of transport (i.e., “Car” and “Underground”), while for the summer period, no statistically significant differences were found between the MEs considered.

## Figures and Tables

**Table 1:** Summary of the micro-environments (MEs) considered in this study. Hour and time of stay refers to those a priori planned, even if small variations should be considered. (LT: low-traffic condition; HT: high-traffic condition; n.a.: not available). * Return trip—these MEs refer to the same MEs frequented during the first part of the journey.

ME	Hour (From–To; min)	Time of Stay (min)	Route Length (km)
Car	7:50–8:10	20	10
Walking (LT)	8:25–8:35	10	0.7
Train	8:45–9:35	50	45
Walking (LT)	9:35–9:55	20	1.5
Walking (HT)	9:55–10:05	10	0.5
Underground	10:05–10:15	10	2.5
Walking (HT)	10:20–10:30	10	0.6
Cycling	10:30–10:50	20	3
Indoor	10:50–12:00	70	n.a
Walking (HT) *	12:00–12:10	10	0.6
Underground *	12:10–12:20	10	2.5
Walking (HT) *	12:20–12:30	10	0.5
Walking (LT) *	12:30–12:50	20	1.5
Train *	13:20–14:10	50	45
Walking (LT) *	14:10–14:20	10	0.7
Car *	14:20–14:40	20	10

**Table 2:** Particulate matter (PM) mass values (µg) deposited in the respiratory tract during the monitoring period (8:00 a.m. to 3:00 p.m.) (sections: H: head; TB: tracheobronchial; P: pulmonary; total: H + TB + P).

Season	Pollutant	H	TB	P	Total
Summer	PM_1_	0.76	0.29	0.57	1.62
	PM_2.5_	3.17	0.56	1.35	5.07
	PM_4_	5.60	0.74	1.26	7.60
	PM_10_	11.24	0.41	0.08	11.73
Winter	PM_1_	0.87	0.33	0.66	1.87
	PM_2.5_	3.94	0.69	1.68	6.31
	PM_4_	7.29	0.96	1.64	9.89
	PM_10_	15.80	0.58	0.12	16.50

**Table 3:** PM deposited mass (µg) in the respiratory tract (H: head; TB: tracheobronchial; P: pulmonary; total: H + TB + P) estimated across the micro-environments (MEs) visited by the commuter.

	ME	Winter				Summer			
		Head	TB	P	Total	Head	TB	P	Total
PM_1_	Walking (LT)	0.284	0.108	0.215	0.607	0.356	0.136	0.270	0.762
	Walking (HT)	1.381	0.528	1.046	2.955	1.574	0.602	1.192	3.368
	Bike	0.666	0.255	0.505	1.426	0.465	0.178	0.352	0.994
	Car	0.231	0.088	0.175	0.495	0.136	0.052	0.103	0.291
	Underground	2.155	0.824	1.632	4.611	0.636	0.243	0.481	1.360
	Train	0.615	0.235	0.466	1.316	0.537	0.205	0.407	1.148
	Indoor	0.918	0.351	0.695	1.964	0.852	0.326	0.646	1.824
	Other	2.479	0.948	1.878	5.305	1.979	0.757	1.499	4.235
PM_2.5_	Walking (LT)	1.233	0.217	0.524	1.975	1.400	0.246	0.595	2.241
	Walking (HT)	6.039	1.061	2.568	9.668	6.256	1.099	2.660	10.016
	Bike	2.961	0.520	1.259	4.741	1.929	0.339	0.820	3.088
	Car	0.923	0.162	0.393	1.478	0.553	0.097	0.235	0.885
	Underground	10.966	1.927	4.662	17.555	3.207	0.563	1.363	5.134
	Train	2.496	0.439	1.061	3.995	2.021	0.355	0.859	3.236
	Indoor	4.048	0.711	1.721	6.480	3.427	0.602	1.457	5.486
	Other	11.023	1.937	4.687	17.647	8.702	1.529	3.700	13.930
PM_4_	Walking (LT)	2.189	0.287	0.494	2.971	2.454	0.322	0.554	3.329
	Walking (HT)	11.457	1.504	2.585	15.546	11.028	1.448	2.489	14.964
	Bike	5.814	0.763	1.312	7.889	3.509	0.461	0.792	4.761
	Car	1.510	0.198	0.341	2.049	0.930	0.122	0.210	1.261
	Underground	21.110	2.771	4.764	28.644	6.103	0.801	1.377	8.281
	Train	4.311	0.566	0.973	5.850	3.337	0.438	0.753	4.528
	Indoor	7.425	0.975	1.676	10.076	5.968	0.783	1.347	8.099
	Other	20.145	2.644	4.546	27.335	15.728	2.065	3.549	21.342
PM_10_	Walking (LT)	5.857	0.214	0.043	6.114	5.355	0.196	0.039	5.590
	Walking (HT)	26.306	0.963	0.193	27.462	22.228	0.814	0.163	23.204
	Bike	13.825	0.506	0.101	14.432	7.196	0.263	0.053	7.513
	Car	2.524	0.092	0.018	2.635	1.609	0.059	0.012	1.679
	Underground	44.346	1.624	0.325	46.295	12.666	0.464	0.093	13.223
	Train	8.973	0.329	0.066	9.367	6.418	0.235	0.047	6.700
	Indoor	16.018	0.586	0.117	16.722	11.533	0.422	0.084	12.039
	Other	42.550	1.558	0.312	44.420	31.820	1.165	0.233	33.218

**Table 4:** Estimated values of the inhaled dose (µg) for the different fractions of PM, divided by the ME considered, time spent commuting, gender, and monitoring period. The colors qualitatively indicate the increase in the doses of inhaled pollutants (from green—lower inhaled doses, to red—higher inhaled doses).

				Summer		Winter						Summer		Winter	
PM_1_	**ME**	**Time (min)**	**Gender**	**Morning**	**Afternoon**	**Morning**	**Afternoon**	PM_2.5_	**ME**	**Time (min)**	**Gender**	**Morning**	**Afternoon**	**Morning**	**Afternoon**
	Train	15	Female	2843	5330	3782	7379		Train	15	Female	3549	6341	5078	9283
		30		5685	10,660	7564	14,758			30		7098	12,681	10,155	18,565
		60		11,370	21,320	15,129	29,515			60		14,197	25,362	20,311	37,131
		90		17,055	31,980	22,693	44,273			90		21,295	38,044	30,466	55,696
		15	Male	3503	6568	4661	9093			15	Male	4374	7813	6257	11,439
		30		7005	13,136	9321	18,185			30		8747	15,626	12,514	22,877
		60		14,011	26,272	18,643	36,371			60		17,494	31,253	25,028	45,754
		90		21,016	39,408	27,964	54,556			90		26,241	46,879	37,542	68,632
	Underground	15	Female	5126	5830	4162	2832		Underground	15	Female	6224	7136	5257	13,360
		30		10,251	11,660	8323	5663			30		12,447	14,273	10,515	26,720
		60		20,503	23,320	16,646	11,327			60		24,895	28,546	21,030	53,441
		90		30,754	34,979	24,969	16,990			90		37,342	42,819	31,545	80,161
		15	Male	6316	7184	5128	3489			15	Male	7669	8794	6479	4542
		30		12,632	14,368	10,256	6979			30		15,338	17,588	12,957	9083
		60		25,265	28,736	20,513	13,957			60		30,677	35,176	25,914	18,167
		90		37,897	43,104	30,769	20,936			90		46,015	52,764	38,871	27,250
	Car	15	Female	5507	3244	4446	3331		Car	15	Female	7172	3838	5149	11,009
		30		11,015	6489	8892	6662			30		14,343	7675	10,297	22,019
		60		22,030	12,977	17,784	13,324			60		28,687	15,351	20,594	44,037
		90		33,044	19,466	26,677	19,986			90		43,030	23,026	30,892	66,056
		15	Male	6787	3998	5479	4105			15	Male	8837	4729	6344	5957
		30		13,573	7996	10,957	8209			30		17,675	9458	12,689	11,914
		60		27,146	15,991	21,915	16,419			60		35,350	18,916	25,378	23,827
		90		40,719	23,987	32,872	24,628			90		53,024	28,374	38,067	35,741
	Bicycle	15	Female	9962	3920	10,387	5714		Bicycle	15	Female	11,707	6197	13,561	17,905
		30		19,924	7839	20,774	11,428			30		23,414	12,395	27,122	35,809
		60		39,848	15,678	41,548	22,856			60		46,828	24,790	54,244	71,618
		90		59,772	23,518	62,323	34,283			90		70,243	37,185	81,366	107,427
		15	Male	11,495	4523	11,985	6593			15	Male	13,508	7151	15,647	9498
		30		22,989	9045	23,970	13,186			30		27,016	14,302	31,295	18,996
		60		45,978	18,090	47,941	26,372			60		54,033	28,604	62,589	37,993
		90		68,967	27,136	71,911	39,558			90		81,049	42,905	93,884	56,989
	Walking	15	Female	7655	9264	9348	13,462		Walking	15	Female	9211	11,091	11,867	20,302
		30		15,310	18,529	18,697	26,924			30		18,422	22,182	23,734	40,604
		60		30,619	37,058	37,394	53,847			60		36,845	44,364	47,468	81,209
		90		45,929	55,587	56,091	80,771			90		55,267	66,546	71,202	121,813
		15	Male	8832	10,690	10,787	15,533			15	Male	10,628	12,797	13,693	21,170
		30		17,665	21,379	21,573	31,066			30		21,257	25,595	27,385	42,341
		60		35,330	42,759	43,147	62,131			60		42,513	51,189	54,771	84,682
		90		52,994	64,138	64,720	93,197			90		63,770	76,784	82,156	127,023
	Train	15	Female	4351	7601	6368	11,193		Train	15	Female	6470	10,662	10,044	16,066
		30		8701	15,201	12,737	22,387			30		12,941	21,325	20,088	32,132
		60		17,403	30,403	25,474	44,774			60		25,881	42,649	40,177	64,265
		90		26,104	45,604	38,211	67,161			90		38,822	63,974	60,265	96,397
		15	Male	5361	9366	7848	13,793			15	Male	7973	13,139	12,377	19,798
		30		10,722	18,732	15,695	27,587			30		15,946	26,277	24,754	39,595
		60		21,445	37,464	31,390	55,173			60		31,892	52,555	49,508	79,191
		90		32,167	56,196	47,085	82,760			90		47,838	78,832	74,262	118,786
	Underground	15	Female	7390	8727	6633	4788		Underground	15	Female	10,779	12,295	10,163	7427
		30		14,780	17,454	13,265	9576			30		21,558	24,590	20,325	14,854
		60		29,561	34,909	26,531	19,152			60		43,116	49,180	40,651	29,708
		90		44,341	52,363	39,796	28,727			90		64,674	73,771	60,976	44,562
		15	Male	9107	10,754	8173	5900			15	Male	13,282	15,151	12,523	9152
		30		18,213	21,508	16,346	11,800			30		26,565	30,301	25,046	18,304
		60		36,426	43,017	32,693	23,600			60		53,130	60,603	50,092	36,608
		90		54,640	64,525	49,039	35,399			90		79,695	90,904	75,138	54,911
	Car	15	Female	8760	4562	5839	6100		Car	15	Female	13,319	6527	8063	10,907
		30		17,519	9124	11,677	12,201			30		26,638	13,053	16,126	21,814
		60		35,038	18,248	23,354	24,401			60		53,276	26,107	32,252	43,628
		90		52,558	27,372	35,031	36,602			90		79,914	39,160	48,378	65,442
		15	Male	10,794	5622	7195	7517			15	Male	16,412	8043	9936	13,440
		30		21,588	11,243	14,389	15,034			30		32,825	16,085	19,871	26,881
		60		43,176	22,486	28,779	30,069			60		65,650	32,170	39,743	53,761
		90		64,764	33,729	43,168	45,103			90		98,475	48,256	59,614	80,642
	Bicycle	15	Female	14,062	7996	17,024	10,279		Bicycle	15	Female	20,648	12,282	26,190	16,137
		30		28,123	15,992	34,048	20,558			30		41,296	24,564	52,379	32,274
		60		56,247	31,983	68,096	41,116			60		82,591	49,128	104,759	64,548
		90		84,370	47,975	102,145	61,674			90		123,887	73,692	157,138	96,822
		15	Male	16,225	9226	19,643	11,860			15	Male	23,824	14,172	30,219	18,620
		30		32,450	18,452	39,286	23,721			30		47,649	28,343	60,438	37,239
		60		64,900	36,904	78,573	47,441			60		95,298	56,686	120,876	74,479
		90		97,350	55,356	117,859	71,162			90		142,947	85,029	181,314	111,718
	Walking	15	Female	11,171	13,077	14,773	22,466		Walking	15	Female	16,402	17,652	23,075	32,460
		30		22,341	26,155	29,545	44,933			30		32,803	35,304	46,150	64,920
		60		44,682	52,310	59,091	89,866			60		65,607	70,607	92,301	129,840
		90		67,023	78,465	88,636	134,799			90		98,410	105,911	138,451	194,760
		15	Male	12,889	15,089	17,045	25,923			15	Male	18,925	20,368	26,625	37,454
		30		25,778	30,179	34,091	51,846			30		37,850	40,735	53,250	74,908
		60		51,556	60,357	68,181	103,691			60		75,700	81,470	106,501	149,815
		90		77,335	90,536	102,272	155,537			90		113,550	122,205	159,751	224,723

**Table 5:** Mann–Whitney *U* test significance values. *p* values of <0.005 are highlighted in red.

Comparison between MEs		Train	Underground	Car	Cycling	Walking
PM_1_	Train		0.747	0.555	0.058	0.001
	Underground			0.658	0.043	<0.001
	Car				0.018	<0.001
	Cycling					0.136
	Walking					
PM_2.5_	Train		0.573	1.000	0.008	0.001
	Underground			0.582	0.023	0.003
	Car				0.006	0.001
	Cycling					0.444
	Walking					
PM_4_	Train		0.872	0.502	0.014	0.001
	Underground			0.658	0.009	<0.001
	Car				0.003	<0.001
	Cycling					0.271
	Walking					
PM_10_	Train		0.936	0.809	0.011	0.001
	Underground			0.799	0.007	<0.001
	Car				0.003	<0.001
	Cycling					0.340
	Walking					

## Supplementary Materials

The following are available online at https://www.mdpi.com/1660-4601/17/17/6066/s1, Materials and Methods—integration to the text; Table S1: Mann–Whitney U test significance values for the comparison between different micro-environments during summer and during winter. *p* values of <0.005 are highlighted in red.
