# Supplementary material for: Estimation of the Inhaled Dose of Airborne Pollutants during Commuting: Case Study and Application for the General Population
Source: Int J Environ Res Public Health. 2020 Aug 20;17(17):6066. doi: 10.3390/ijerph17176066 (PMC7504492; doi:10.3390/ijerph17176066)
Supplement: Supplementary file 1 [file ijerph-17-06066-s001.pdf]

International Journal of
Environmental Research and Public Health

MDPI

Supplementary Material

Estimation of the Inhaled Dose of Airborne Pollutants during Commuting: Case Study and Application for the General Population

Francesca Borghi *, Giacomo Fanti *, Andrea Cattaneo, Davide Campagnolo, Sabrina Rovelli, Marta Keller, Andrea Spinazzè and Domenico Maria Cavallo

Department of Science and High Technology, University of Insubria, 22100, Como, Italy; andrea.cattaneo@uninsubria.it (A.C.); davide.campagnolo@uninsubria.it (D.C.); sabrina.rovelli@uninsubria.it (S.R.); mkeller@studenti.uninsubria.it (M.K.); andrea.spinazze@uninsubria.it (A.S.); domenico.cavallo@uninsubria.it (D.M.C.)
* Correspondence: f.borghi2@uninsubria.it (F.B.); g.fanti@studenti.uninsubria.it (G.F.); Tel.: +39 031 238 6645

Materials and Methods—integration to the text

To integrate the text, the materials and methods already used in Borghi and collaborators (Borghi et al., 2020) and used in this study are reported below.

Study Design and Instrumentation

To simulate a typical home-to-work (and return) commuter's route, a fixed route (for a total of 90 km) was defined a priori from a provincial city ('home' (Villa Guardia), 45° 47′ N 9° 01′ E) to an office located in Milan ('Workplace', 45° 27′ N 9° 11′ E), the largest city in Lombardy, Italy.

With the use of a commuting route, different MEs usually visited by commuters were considered: the MEs visited by the commuter were as follows: walking (low traffic (LT) condition), Walking (high traffic (HT) condition), Bike, Car, Underground, Train, Indoor, and Other MEs (defined as the transition period (2 min) between an environment to another). Experimental data were collected over two working weeks (Monday to Friday) in two different seasons (winter campaign, 11 March 2019–15 March 2019 and 18 March 2019–22 March 2019; summer campaign, 8 July 2019–12 July 19*, 15 July 2019–19 July 19; *the monitoring on Thursday (11 July 2019) was cancelled due to a public transport strike and was re-scheduled the following available Thursday (25 July 2019)) to characterize the weekly and seasonal pollutants' concentration variability.

Portable and miniaturized monitors were used to assess the exposure levels to different airborne pollutants. All the instruments were worn by one of the authors (F.G.) using a backpack. All instrument inlets were placed in the breathing zone of the operator, with the 30 cm-radius hemisphere extending in front of the face. All instruments were checked daily, and all guidelines provided by the manufacturer were followed to ensure quality-controlled data. Instruments were also constantly checked during the monitoring phase to prevent instrument failure. All instruments were set up with an acquisition rate equal to 60 s.

Different portable instruments, both direct-reading and filter-based, were used to evaluate size-fractionated PM exposure. UFP exposure levels were measured using a portable diffusion size classifier (DiSCmini (DSC), Matter Aerosol AG, Wohlen AG, Swiss). The DSC used in this study can measure the number concentration and the average size of the particles in the range of 10 < Dp < 700 nm. The continuous determination of size-fractionated PM

concentration was also performed using a second portable direct-reading monitor (Aerocet 831-Met One Instrument Inc., Grant Pass, Oregon, USA), which provides the concentration data of the different PM fractions (PM_1, $PM_{2.5}$, PM_4, PM_{10}, and TSP). Finally, a complementary miniaturized monitor was used for the evaluation of $PM_{2.5}$ concentration (AirBeam (AB), HabitatMap Inc., Brooklyn, New York, USA). This monitor is based on an Arduino board, and it can detect particles in a range from 0.5 to 2.5 μm and a $PM_{2.5}$ concentration up to 400 μg/m^3. $PM_{2.5}$ samples were collected using a GK2.05 sampler (BGI Inc., Waltham, MA, USA), operated with a sampling pump with a flow rate equal to 4 L/min; the particles were collected using polytetrafluoroethylene filters. Mass concentration was determined by performing gravimetric analysis following a standard reference method (12341, 2014). The weighing procedure (Spinazzè et al., 2017; Borghi et al., 2018) considered the conditioning of the filters in a controlled environment (temperature (T), 20 ± 1 °C; relative humidity (RH), 50±5%) for a minimum of 24 h. Subsequently, the filters were weighted before and after the sampling using a microbalance (Gibertini Micro 1000, Novate, Milan, Italy). Gravimetric data were used to correct the PM data outcomes from the direct-reading instruments, providing a daily correction factor, applied a posteriori to the whole PM dataset.

The measurement of NO_2 concentration was performed using a miniaturized electrochemical monitor (CairClip NO_2, Cairpol; La Roche Blanche, France). The subject's heart rate was measured using a heart rate monitor (Suunto 9). This instrument was also used to acquire Global Positioning System data, with the same acquisition rate to that of other used instruments (60 s).

Statistical Analysis and Inhaled Dose Estimation

Following the well established practices in statistics and the literature, data obtained using direct-reading instruments were examined and handled to exclude zero and unreliable data: for this reason, concentration distributions were truncated above the 99th percentile and below the first percentile (Hänninen et al., 2003). Moreover, following the literature (Spinelle, Gerboles and Aleixandre, 2015) on the validation and evaluation of micro-sensors, an NO_2 value below the calculated limit of detection (LOD) ('LOD' = 1.692 μg/m^3) was replaced with LOD/2. Furthermore, following the technical references of the direct-reading instruments, the PM data obtained in extreme microclimatic conditions (RH > 80%; T > 50 °C) were eliminated to exclude the data afflicted by recognized environmental interference. As mentioned previously, the error associated with the PM direct-reading instruments was managed using a calculated correction factor. The correction factor, calculated by dividing daily PM concentration measured gravimetrically with the daily average PM concentration measured simultaneously using direct-reading instruments, was applied to the data measured from direct-reading instrument monitoring (Jenkins et al., 2004; Spinazzè et al., 2017). UFP mass concentrations were calculated based on the number of concentrations, particle diameter, and mean mass density factors.

As reported in the literature (Tan, Roth and Velasco, 2017), the pollutant inhaled dose can be estimated as the product of the measured exposure concentration, the ventilation rate, and the time spent in each specific ME. In this regard, the subject's ventilation rate was calculated following the literature (Dias Do Vale, 2014), where the ventilation rate (l/min) was calculated as reported in Equation 1, considering the heart rate (bpm) of the subject. The descriptive statistic of the inhaled dose was reported in this study as the average dose calculated in each ME:

$$VE = 0.00071 \times HR2.17 \quad (1)$$

Equation 1. Calculation of the ventilation rate (Dias Do Vale, 2014). VE: ventilation rate (l/min); HR: heart rate (bpm).

References

12341, U. E. (2014) 'Ambient air. Standard gravimetric measurement method for the determination of the PM10 or PM2,5 mass concentration of suspended particulate matter.'

Borghi, F. *et al.* (2018) 'Precision and accuracy of a direct-reading miniaturized monitor in PM2.5 exposure assessment', *Sensors (Switzerland)*, 18(9), pp. 1–21. doi: 10.3390/s18093089.

Borghi, F. *et al.* (2020) 'Commuters' Personal Exposure Assessment and Evaluation of Inhaled Dose to Different Atmospheric Pollutants', *International Journal of Environmental Research and Public Health*, 17(10), p. 3357. doi: 10.3390/ijerph17103357.

Dias Do Vale, I. (2014) 'Comparison of pedestrians' particulate matter inhalation for different routes in urban centers Environmental Engineering Examination Comittee', (June). Available at: https://fenix.tecnico.ulisboa.pt/downloadFile/281870113701912/INES DO VALE dissertacao de mestrado.pdf.

Hänninen, O. *et al.* (2003) 'EXPOLIS simulation model: PM2.5 application and comparison with measurements in Helsinki', *Journal of Exposure Analysis and Environmental Epidemiology*, 13(1), pp. 74–85. doi: 10.1038/sj.jea.7500260.

Jenkins, R. A. *et al.* (2004) 'Development and application of protocols for the determination of response of real-time particle monitors to common indoor aerosols', *Journal of the Air and Waste Management Association*, 54(2), pp. 229–241. doi: 10.1080/10473289.2004.10470892.

Spinazzè, A. *et al.* (2017) 'Field comparison of instruments for exposure assessment of airborne ultrafine particles and particulate matter', *Atmospheric Environment*, 154, pp. 274–284. doi: 10.1016/j.atmosenv.2017.01.054.

Spinelle, L., Gerboles, M. and Aleixandre, M. (2015) 'Performance evaluation of amperometric sensors for the monitoring of O3 and NO2 in ambient air at ppb level', *Procedia Engineering*, 120, pp. 480–483. doi: 10.1016/j.proeng.2015.08.676.

Tan, S. H., Roth, M. and Velasco, E. (2017) 'Particle exposure and inhaled dose during commuting in Singapore', *Atmospheric Environment*. Elsevier Ltd, 170, pp. 245–258. doi: 10.1016/j.atmosenv.2017.09.056.

Table S1. Mann–Whitney U test significance values for the comparison between different micro-environments during summer and during winter. *p* values of <0.005 are highlighted in red.

	**Summer**							**Winter**					
		Train	Underground	Car	Cycling	Walking			Train	Underground	Car	Cycling	Walking
PM_1	Train		0.243	0.734	0.152	0.013	PM_1	Train		0.142	0.243	0.243	0.014
	Underground			0.366	0.821	0.122		Underground			0.706	0.009	0.001
	Car				0.274	0.026		Car				0.026	0.001
	Cycling					0.429		Cycling					0.187
	Walking							Walking					
		Train	Underground	Car	Cycling	Walking			Train	Underground	Car	Cycling	Walking
$PM_{2.5}$	Train		0.243	0.624	0.090	0.016	$PM_{2.5}$	Train		0.734	0.624	0.046	0.012
	Underground			0.522	0.522	0.132		Underground			0.940	0.019	0.005
	Car				0.175	0.032		Car				0.022	0.006
	Cycling					0.678		Cycling					0.498
	Walking							Walking					
		Train	Underground	Car	Cycling	Walking			Train	Underground	Car	Cycling	Walking
PM_4	Train		0.243	0.624	0.060	0.019	PM_4	Train		0.175	0.187	0.152	0.012
	Underground			0.522	0.346	0.132		Underground			0.821	0.007	0.001
	Car				0.113	0.035		Car				0.013	0.001
	Cycling					0.763		Cycling					0.228
	Walking							Walking					
		Train	Underground	Car	Cycling	Walking			Train	Underground	Car	Cycling	Walking
PM_{10}	Train		0.187	0.498	0.042	0.019	PM_{10}	Train		0.200	0.291	0.105	0.013
	Underground			0.598	0.346	0.142		Underground			0.763	0.007	0.001
	Car				0.113	0.042		Car				0.013	0.002
	Cycling					0.940		Cycling					0.243
	Walking							Walking					
